# Surge of Sympathetic Activity during Hyperventilation at the End of Apnea for Patients with Obstructive Sleep Apnea

**DOI:** 10.3390/medicina60030366

**Published:** 2024-02-22

**Authors:** Jui-Kun Chiang, Yen-Chang Lin, Hsueh-Hsin Kao, Yee-Hsin Kao

**Affiliations:** 1 Department of Family Medicine, Dalin Tzu Chi Hospital, Buddhist Tzu Chi Medical Foundation, No. 2, Minsheng Road, Dalin, Chiayi 622, Taiwan; roma@tzuchi.com.tw; 2 Nature Dental Clinic, Nantou 545, Taiwan; drlin@alliswell.tw; 3 Department of Radiation Oncology, Taichung Veterans General Hospital, Taichung 407, Taiwan; 4 Department of Family Medicine, Tainan Municipal Hospital (Managed by Show Chwan Medical Care Corporation), 670 Chung-Te Road, Tainan 701, Taiwan

**Keywords:** sympathetic activity, hyperventilation, heart rate variability (HRV), obstructive sleep apnea (OSA), polysomnography (PSG)

## Abstract

*Background and Objectives*: The mechanisms connecting obstructive sleep apnea (OSA) and cardiovascular disease are multifactorial, involving intermittent hypoxia, hypercapnia, and sympathetic activation. The aim of this study was to explore the oscillations of sympathetic activity during the sleep apnea episodes throughout the entire night in patients with OSA. *Materials and Methods*: The participants received whole-night polysomnography (PSG), and electrocardiogram (EKG) data from the PSG were collected for heart rate variability (HRV) analysis. HRV measurements were conducted in the time and frequency domains. The root mean square of successive differences between normal heartbeats (RMSSD), which reflects parasympathetic activity, and the ratio of the absolute power of the low-frequency band (0.04–0.15 Hz) to the absolute power of the high-frequency band (0.015–0.4 Hz) (LF/HF ratio), which indicates sympathetic activity, were computed. *Results*: A total of 43 participants (35 men and 8 women) were included in the analysis. The mean age of the participants was 44.1 ± 11.3 years old, and the mean BMI was 28.6 ± 5.4 kg/m^2^. The sleep apnea episodes throughout the entire night in patients with OSA were selected randomly and occurred most frequently during the non-REM stages (39, 90.7%). The selected sleep apnea episodes typically exhibited multiple apneas, often interrupted by snoring respiration and followed by hyperventilation at the end of the episode (HE). Our findings indicate that the centers of the 5 min HRV window for the lowest and highest LF/HF ratios, at 111.8 ± 88.2 and 117.4 ± 88.6 min after sleep onset, respectively, showed a statistically significant difference (*p* < 0.001). Similarly, the ratios of the lowest and highest LF/HF, at 0.82 ± 0.56 and 3.53 ± 2.94, respectively, exhibited a statistically significant difference (*p* < 0.001). *Conclusions*: In the current study, the selected sleep apnea episodes throughout the entire night in patients with OSA occurred primarily during the non-REM stages. Additionally, we observed that sympathetic activity reached its peak in the window that includes hyperventilation at the end stage of apnea, potentially posing a cardiovascular risk. However, additional studies are needed to validate these results.

## 1. Introduction

Obstructive sleep apnea (OSA) is a prevalent disorder that may manifest with or without symptoms, and it is associated with substantial neurocognitive and cardiovascular consequences [1]. OSA induces significant acute hemodynamic changes, and there are suggested causal relationships with hypertension, cardiovascular morbidity, and mortality [2]. According to a previous study, OSA is correlated with a 1.9-fold increase in all-cause mortality and a 2.65-fold increase in cardiovascular mortality [3]. Using the American Academy of Sleep Medicine (AASM) 2012 scoring criteria for identifying OSA, with apnea/hypopnea index (AHI) threshold values of five or more events per hour and 15 or more events per hour, it is estimated that globally, 936 million adults aged 30–69 years have OSA, with 425 million adults aged 30–69 years experiencing moderate to severe OSA. China has the highest number of affected individuals, followed by the USA, Brazil, and India [4]. A cohort study revealed that untreated OSA poses a significant risk for high mortality rates, contributing to both all-cause mortality and cardiovascular mortality [5].

The health impact of OSA involves the generation of exaggerated negative intrathoracic pressure, resulting in increased left ventricular wall tension and a reduction in stroke volume [6]. Additionally, OSA induces a decrease in the activity of upper airway dilator muscles, respiratory arousal [7], apnea threshold, chemosensitivity, cardiovascular function, and alterations in sleep state [8]. The mechanisms underlying the association between OSA and cardiovascular disease are multifactorial and involve intermittent hypoxia, hypercapnia, and sympathetic activation. Numerous studies consistently demonstrate that individuals with OSA exhibit elevated levels of sympathetic nerve activity [9]. Furthermore, by influencing sympathetic neural mechanisms, OSA may contribute to or exacerbate elevated blood pressure levels in a significant portion of the hypertensive patient population. This intricate interplay of physiological factors underscores the complex relationship between OSA and cardiovascular health [10].

A prior study investigated the relationship between sleep apnea/hypopnea and sleep stages, revealing that 82% of patients diagnosed with OSA were classified as having non-rapid eye movement (NREM)-related OSA, according to the broad definition (AHI_REM_/AHI_NREM_ ≥ 2). In contrast, 97.3% of patients met the criteria for the strict definition (AHI_REM_ > 5 and AHI_NREM_ < 5, with a total REM sleep duration of at least 30 min) [11]. Another previous study indicated that a higher AHI, indicative of severe OSA, was observed during non-REM sleep, particularly in the N1 stage [12]. A prior study reported a positive and significant association between a higher log LF/HF power ratio and AHI during whole-night PSG in adult patients [13]. Accordingly, the majority of OSA incidents were found to be associated with non-REM sleep. Although non-REM sleep constitutes approximately 75 to 80 percent of the total time spent in sleep, with REM sleep comprising the remaining 20 to 25 percent, in normal subjects, there is a rise in sympathetic activity during REM sleep [14]. Sympathetic nerve activity decreases as non-REM sleep deepens in normal subjects; however, during non-REM sleep, there is a burst of sympathetic nerve activity due to the brief increase in blood pressure and heart rate following K-complexes (arousal stimuli during stage 2 sleep elicited high-amplitude deflections on the electroencephalogram, called K complexes) [14]. The impact of sympathetic activity during non-REM sleep for patients with OSA represents an intriguing area for investigation.

OSA is characterized by the recurrent collapse of the upper airway during sleep, leading to significantly reduced (hypopnea) or completely absent (apnea) ventilation despite ongoing respiratory efforts [8]. This results in inadequate ventilation to meet the body’s requirements, causing hypoxemia and hypercapnia, which further stimulate respiratory effort. However, this increased drive is often ineffective in increasing ventilation without spontaneous airway opening. Consequently, apnea/hypopnea typically continues until the patient arouses from sleep and terminates the obstruction. Following airway re-opening, hyperventilation occurs to reverse the blood gas disturbances that developed during the respiratory event [8]. However, the influence of sympathetic activity during non-REM sleep is seldom explored, making an investigation into its effects on patients with OSA an intriguing endeavor. This research is further motivated by the observation that the majority of OSA incidents are linked to non-REM sleep. Another previous study noted that the zenith of sympathetic activity occurs during the final 10 s of each apneic event in individuals with OSA [9]. The aim of this study is to investigate the timing of heightened sympathetic activity during the non-REM sleep stage and its association with the duration of snoring, apnea, and subsequent hyperventilation in adult patients with OSA.

## 2. Materials and Methods

### 2.1. Settings and Participants

The participants were referred from the outpatient department to the sleep center of a localized teaching hospital in southern Taiwan due to clinical suspicion of obstructive sleep apnea (OSA) and subsequently underwent whole-night polysomnography (PSG). Most participants were referred from the Ear, Nose, and Throat (ENT) and Internal Medicine departments in the identical hospital between July 2020 and June 2021. Inclusion criteria involved participants who received PSG due to snoring or the severity of OSA, with an Apnea-Hypopnea Index (AHI) greater than 5. Exclusions comprised individuals under the age of 20 and those with severe cardiovascular disorders, severe neuromuscular disorders, a history of surgery for snoring and sleep apnea, or those taking medications that influence the sympathetic nervous system (such as alpha or beta-blockers, and centrally acting drugs).

Informed consent was obtained from all participants before their enrollment in the study. This current study protocol was reviewed/approved by the institutional review board of the Tainan Municipal Hospital (Managed by Show Chwan Medical Care Corporation) (SCMH_IRB No: 1090508).

### 2.2. PSG and Heart Rate Variability (HRV) Measurement

Participants underwent polysomnography (PSG) using the EMBLA N7000 system (Embla Inc., Broomfield, CO, USA). The PSG system was capable of gathering electrophysiological signals for heart activity analysis, pulse oximetry for oxygen saturation measurement, airflow through nasal pressure and oronasal thermal sensors, body position tracking, actigraphy data, and monitoring thoracic motions [15].

Electrocardiogram (EKG) data obtained via the polysomnography (PSG) were extracted for heart rate variability (HRV) analysis. HRV measurements were conducted in both the time and frequency domains, encompassing the root mean square of successive differences between normal heartbeats (RMSSD) and the ratio of the absolute power of the low-frequency (LF) band (0.04–0.15 Hz) to the absolute power of the high-frequency (HF) band (0.15–0.4 Hz) (LF/HF ratio), respectively. RMSSD was acknowledged as a marker of parasympathetic activity [16]. The LF/HF ratio served as an indicator of sympathovagal balance [17]. In this study, the LF/HF ratio was employed to assess sympathetic activity, as per the methodology outlined in the study by AlQatari et al. [18].

### 2.3. Define Typical Apnea Episodes and the Timing of Lowest and Highest Sympathetic Activity

Sleep apnea episodes throughout the entire night were randomly selected. The event criteria for assessing sleep hypopnea episodes involved the comparison of O_2_ saturation, specifically focusing on instances where it fell below 90% and lasted for ≥10 s, in conjunction with monitoring nasal flow patterns. A typical sleep apnea episode exhibited multiple apneas, often interrupted by snoring respiration and followed by hyperventilation at the end of the episode (HE). The HE may encompass the process from arousal to the reopening of the airway and subsequent hyperventilation at the end of the apnea episode.

The HRV was calculated using a time window of 5 min and shifted by 10 s along the sleep apnea episodes. Within the sleep apnea episodes, which typically exceeded 5 min in duration, the lowest LF/HF ratios were observed. Specifically, the window encompassing the hyperventilation at the end of the episode (HE) tended to exhibit the highest LF/HF ratios. 

In this study, the nadir of sympathetic activity within the identified sleep apnea episode was designated as the low group. Additionally, the zenith of sympathetic activity, typically incorporating the HE, particularly following the matched sleep apnea episode, was classified as the high group. The HRV window was calculated to be 5 min, and the centers of the windows for both nadirs and zeniths were defined accordingly.

### 2.4. Statistical Analysis

We utilized the free R software, version 4.0.3 (R Foundation for Statistical Computing, Vienna, Austria), for data analysis. Statistical significance was set at 0.05, and all assessments were two-sided. Continuous measurements were presented as mean ± standard deviation. The Wilcoxon test or *t*-test was applied, as appropriate. Categorical measurements were presented as *n* (%), with Fisher’s exact test applied. Electrocardiogram data were downloaded from the PSG. The seewave, ebm, and RHRV packages with Hilbert transformation were utilized. Data files were visually inspected for artifacts [19]. Paired *t*-tests were employed to evaluate differences between groups. Time series plots of HRV, nasal airflow, and O_2_ saturation were matched to select the sleep apnea episode.

## 3. Results

**Figure 1:**
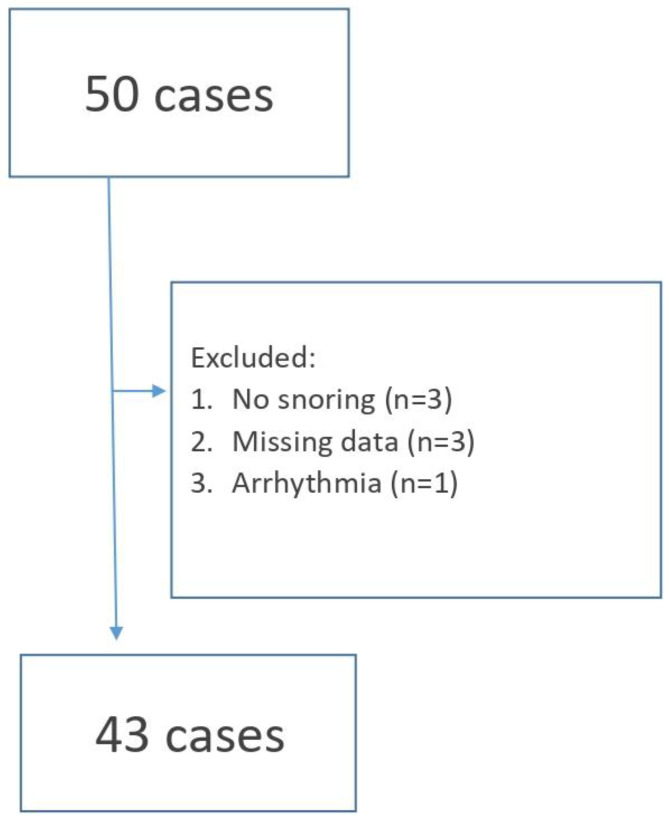
Flow chart of this study.

In the analysis, a total of 43 participants were included, comprising 35 men and 8 women. The participants had a mean age of 44.1 ± 11.3 years and a mean BMI of 28.6 ± 5.4 kg/m^2^. Notably, neck circumference was higher in males (40.7 ± 5.0 cm) compared to females (36.1 ± 3.1 cm), showing a statistically significant difference (*p* = 0.001). The numbers for mild (5 ≤ AHI < 15 events/h), moderate (15 ≤ AHI < 30 events/h), and severe (AHI ≥ 30 events/h) OSA were 12, 12, and 19 patients, respectively. We also observed that randomly selected sleep apnea episodes throughout the entire night in patients with OSA occurred most frequently during the non-REM stages (39, 90.7%) (Table 1). The flow chart of this study was presented in Figure 1. The whole-night RMSSD and LF/HF ratios were 97.2 ± 48.6 and 1.35 ± 0.74, respectively, with no significant gender-based differences.

**Figure 2:**
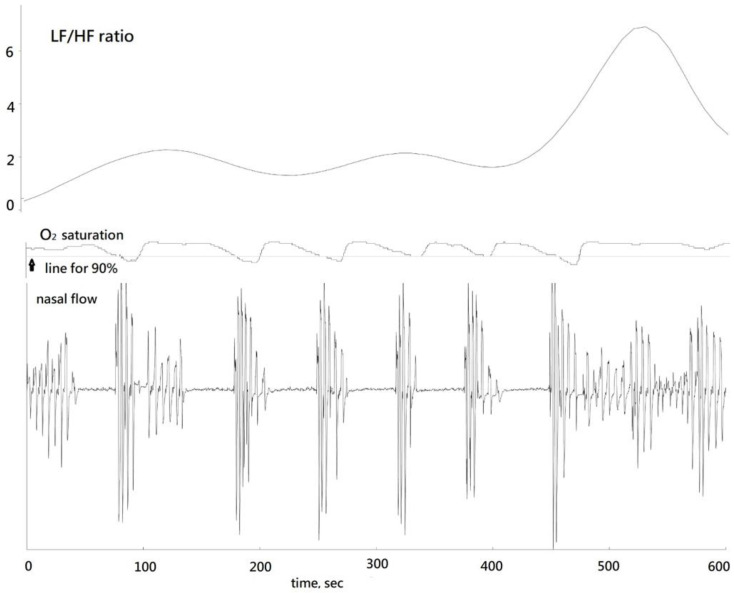
Corresponding LF/HF ratio, oxygen saturation, and nasal flow in a standard sleep apnea episode.

A standard sleep apnea episode manifested multiple apneas, frequently punctuated by snoring respiration subsequent to hyperventilation at the end of the episode (HE), occurring between the 450th and 600th seconds (Figure 2). A decrease in blood oxygen saturation (SPO_2_) levels below 90%, with the lowest recorded level being 87%, was observed.

**Figure 3:**
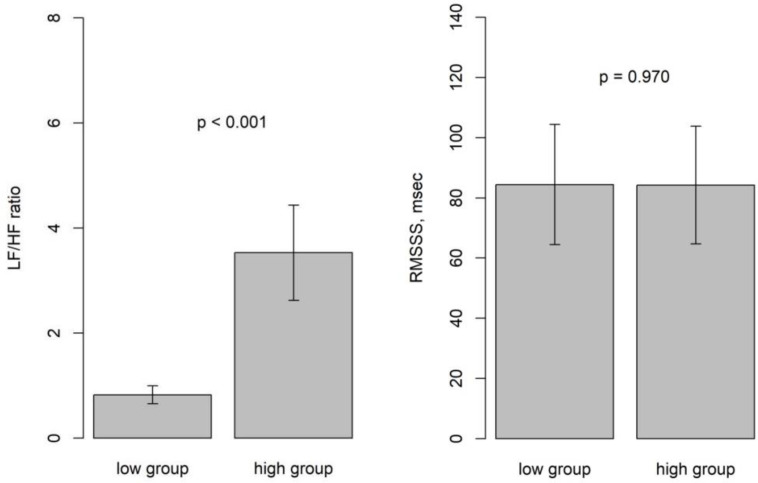
Bar plots depicting LF/HF and RMSSD values for the low and high groups.

The time window for heart rate variability analysis was 5 min and the centers of HRV could be defined accordingly. Moving HRV was calculated by shifting every 10 s. Within the sleep apnea episodes, which usually were longer than 5 min and encompassed several apneas, the lowest LF/HF ratios were observed. The window including the HE had the highest LF/HF ratios. Our findings indicate that the centers of the HRV window for the lowest and highest LF/HF ratios were observed at 111.8 ± 88.2 and 117.4 ± 88.6 min after sleep onset, respectively, with this difference proving statistically significant (*p* < 0.001). The lowest LF/HF ratio was 0.82 ± 0.56 and the highest was 3.53 ± 2.94. The increase in LF/HF ratio was statistically significant (*p* < 0.001). However, RMSSD values did not differ significantly between the low and high LF/HF ratio groups (*p* = 0.970) (Table 2). Bar plots depicted LF/HF and RMSSD values for the low and high groups (Figure 3). Additionally, we investigated the relationship between the time difference from the low group to the high group and the differences in LF/HF ratio between these two groups.

**Figure 4:**
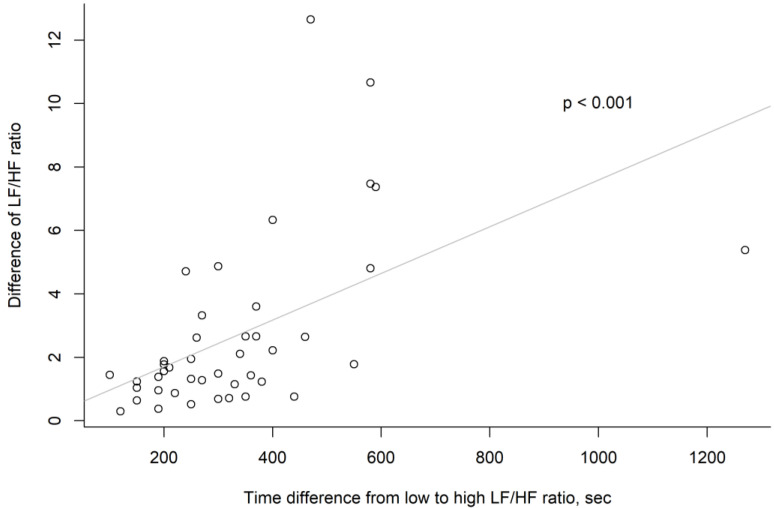
Scatter plot of each analysis point in “time difference from low to high LF/HF ratio” and “difference of LH/HF ratio” vectors.

Our analysis revealed a positive correlation between these factors (*p* < 0.001) (Figure 4).

## 4. Discussion

In the present study, sleep apnea episodes were randomly selected throughout the entire night in patients with OSA. Sympathetic activity increased, with the majority of episodes occurring during the non-REM stages. In the current study, we employed HRV as a calculation method and found that sympathetic nervous system activity fluctuates, with the highest peak occurring during the sleep apnea episode following hyperventilation at the end of the episode in patients with OSA. Additionally, HRV was calculated using a time window of 5 min and shifted by 10 s along the sleep apnea episodes, resulting in more accurate localization. A previous study reported that the extent of nocturnal oxygen desaturation, a significant pathophysiological consequence of OSA, was an independent risk factor for incident atrial fibrillation in individuals under 65 years of age [20]. If additional interventions can be implemented to prevent the surge in sympathetic activity, it may help prevent the complications of OSA.

Obstructive sleep apnea has been linked to various cardiovascular complications, such as hypertension, heart failure, coronary artery disease, arrhythmias, and cardiovascular mortality [21]. Additionally, OSA poses significant risks, including an elevated risk of overweight and obesity, type 2 diabetes, hyperlipidemia, and physical inactivity [22]. The association between OSA and dysregulation of the autonomic nervous system (ANS) has been a subject of debate for years. However, the pathophysiological mechanisms responsible for these alterations are yet to be fully elucidated. Although the mechanisms linking OSA and cardiovascular disease are multifactorial, our current study specifically concentrates on assessing sympathetic activity in patients with OSA. A prior study reported that REM sleep is typically associated with significant sympathetic activation in normal subjects [14]. A previous study reported that the presence of OSA accelerated the “decay” rate of non-REM and REM sleep bouts, leading to instability characterized by shorter bouts and an increased number of stage transitions [23]. In another earlier study, it was reported that 72–97.3% of patients with OSA were classified as having non-REM-related OSA based on both broad and strict definitions [11]. Yet another previous study indicated that higher AHI, signifying severe OSA, occurred during the non-REM stage, especially in N1 [12]. The escalation of sympathetic nerve activity in patients with OSA is ascribed to the hyperventilation at the end of the episode, particularly during non-REM sleep.

In the current study, we also observed the highest LF/HF ratio, indicating increased sympathetic activity, during the sleep apnea episode following hyperventilation at the end of the episode in patients with OSA. The heightened sympathetic activity potentially represents an increased cardiovascular risk. A previous study documented a notable increase in blood pressure among individuals with OSA due to the sympathetic vasoconstrictor response during apneic events. Additionally, the study reported that heightened sympathetic activity was particularly observed at the end of the apnea [24]. Additionally, the longer duration of snoring preceding the hyperventilation at the end period, the higher the elevation of sympathetic activity. This could serve as a contributing factor to arrhythmias leading to sudden cardiac death, including cardiovascular mortality and stroke, offering indirect evidence. Nevertheless, further studies are required to validate these findings.

However, the parasympathetic activity, as measured by RMSSD, did not exhibit a significant change between the low and high groups throughout the entire night in patients with OSA.

In this current study, the participants were referred from ENT and Internal Medicine departments to the sleep unit for clinical suspicion of OSA and subsequently underwent whole-night polysomnography (PSG). During the review of the medical records, we did not find any documentation indicating that the patient had visited the cardiology department. Additionally, upon rechecking the medical charts, we found that 8 patients had hypertension. This discovery represents one of the limitations in the current study. Additionally, there are several other limitations to consider. First, we randomly selected sleep apnea episodes throughout the entire night in patients with OSA, and most patients (39, 90.7%) occurred during the non-REM stages. However, the LF/HF ratio in the REM sleep was not examined. It is challenging to accurately identify the REM stage using HRV, and the duration of REM is variable, ranging from 1 to 5 min to as long as 1 h (10–60 min). Addressing this challenge in the analysis would require further investigation using alternative methods. Secondly, the small *n* number of participants were enrolled in this current study (*n* = 43). This is attributed to the strict inclusion criteria, which required participants to have previously been referred for PSG and diagnosed with OSA (AHI ≥ 5). Consequently, only a limited number of participants were included in this current study. Despite the small sample size, the paired t-test for the LF/HF ratio has already indicated a significant difference. Therefore, increasing the sample size will not alter the outcome. Further large-scale studies are needed to validate these findings. Finally, it was observed that most of the randomly selected apneas were associated with non-REM sleep in the current study, without specifying the exact sleep phase. The relationship between sleep apnea and specific sleep phases warrants further study.

## 5. Conclusions

In the current study, the pathophysiological mechanisms of the autonomic nervous system in patients with OSA involve increased sympathetic activity. During sleep apnea episodes, multiple apneas are often interrupted by snoring respiration, followed by hyperventilation at the end of the episode, the majority of which occur during non-REM sleep. These events do not significantly impact parasympathetic activity.

## Figures and Tables

**Table 1:** Demographic characteristics of participants.

	Total	Female	Male	*p*
Number, *n*	43	8	35	
Age, years	44.1 ± 11.3	48.8 ± 12.1	43.1 ± 11.0	0.201
BMI, kg/m^2^	28.6 ± 5.4	26.1 ± 4.3	29.1 ± 5.5	0.126
Neck circumference, cm	40.7 ± 5.0	36.1 ± 3.1	41.7 ± 4.8	0.001
AHI, events/h	33.9 ± 26.0	21.7 ± 15.2	36.7 ± 27.2	0.175
Whole night LF/HF ratio	1.35 ± 0.74	1.11 ± 0.26	1.41 ± 0.80	0.560
Whole night RMSSD	97.2 ± 48.6	116.9 ± 51.4	92.6 ± 47.5	0.178
The lowest LF/HF ratio during the selected sleep apnea episode was randomized				
The timing of the lowest LF/HF ratio, min	111.8 ± 88.2	149.1 ± 119.1	103.2 ± 79.4	0.365
LF/HF ratio	0.82 ± 0.56	0.68 ± 0.32	0.86 ± 0.60	0.720
RMSSD, msec	84.4 ± 64.9	125.2 ± 88.8	75.1 ± 55.7	0.141
Highest LF/HF ratio just after the matched sleep apnea episode				
The timing of the highest LF/HF ratio, min	117.4 ± 88.6	153.0 ± 120.1	109.2 ± 79.7	0.435
LF/HF ratio	3.53 ± 2.94	2.55 ± 1.44	3.75 ± 3.16	0.585
RMSSD, msec	84.3 ± 63.6	116.4 ± 88.6	76.9 ± 55.5	0.211
Non-REM, *n* (%)	39 (90.7%)	7 (87.5%)	32 (91.4%)	0.730

Abbreviations: AHI: apnea–hypopnea index; BMI: body mass index; HF: absolute power of the high-frequency band (0.015–0.4 Hz); LF: absolute power of the low-frequency band (0.04–0.15 Hz); REM: rapid eye movement; RMSSD: root mean square of successive differences.

**Table 2:** LF/HF ratio and RMSSD values stratified by the low and high groups.

	Low Group	High Group	*p*
location, min	111.8 ± 88.2	117.4 ± 88.6	<0.001
LF/HF ratio	0.82 ± 0.56	3.53 ± 2.94	<0.001
RMSSD, msec	84.4 ± 64.9	84.3 ± 63.6	0.970

Abbreviations: HF: absolute power of the high-frequency band (0.015–0.4 Hz); LF: absolute power of the low-frequency band (0.04–0.15 Hz); RMSSD: root mean square of successive differences.

## Data Availability

The data engendered during and/or analyzed in this current study are not publicly available but can be obtained by request from the corresponding author.
